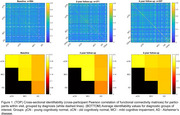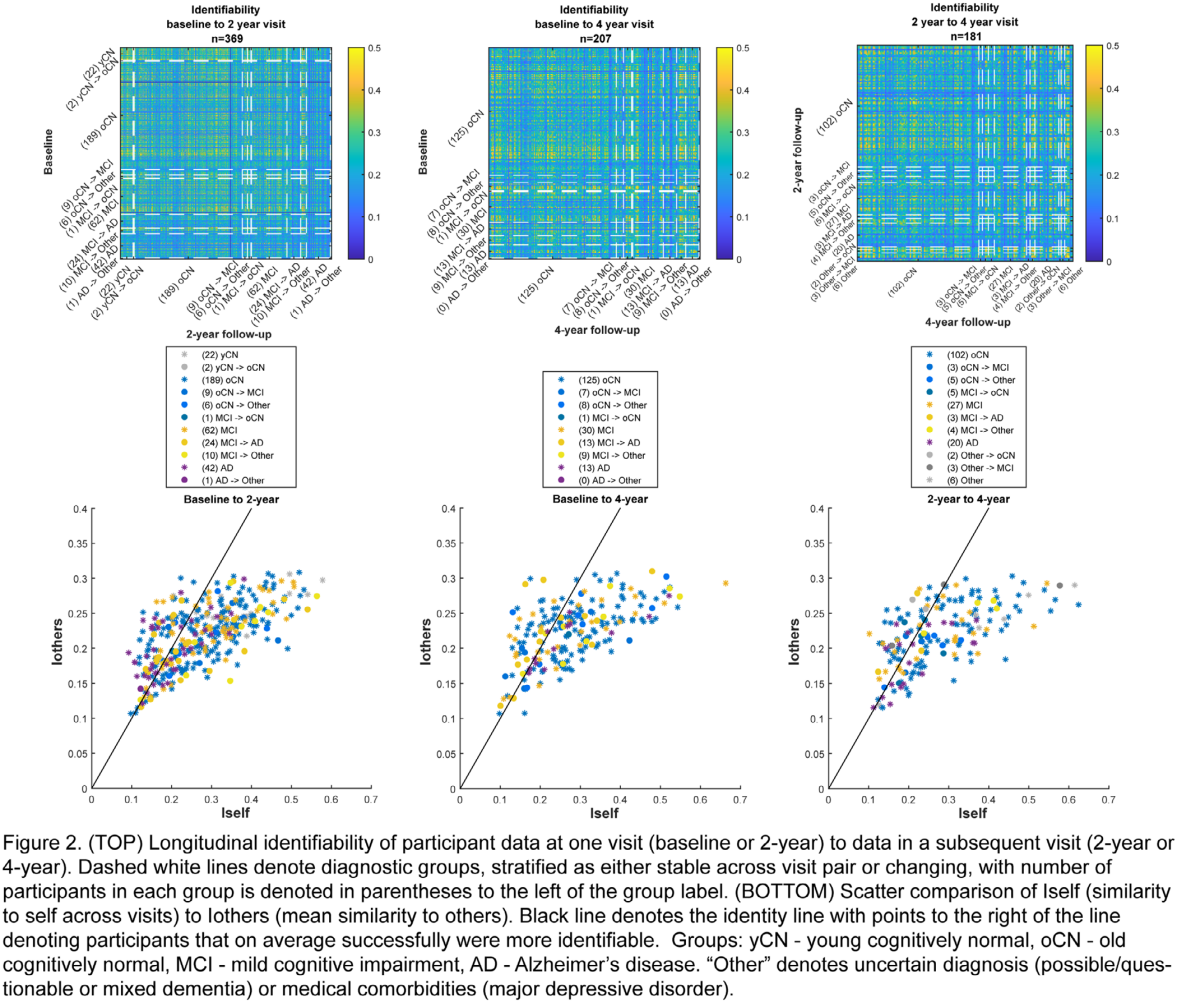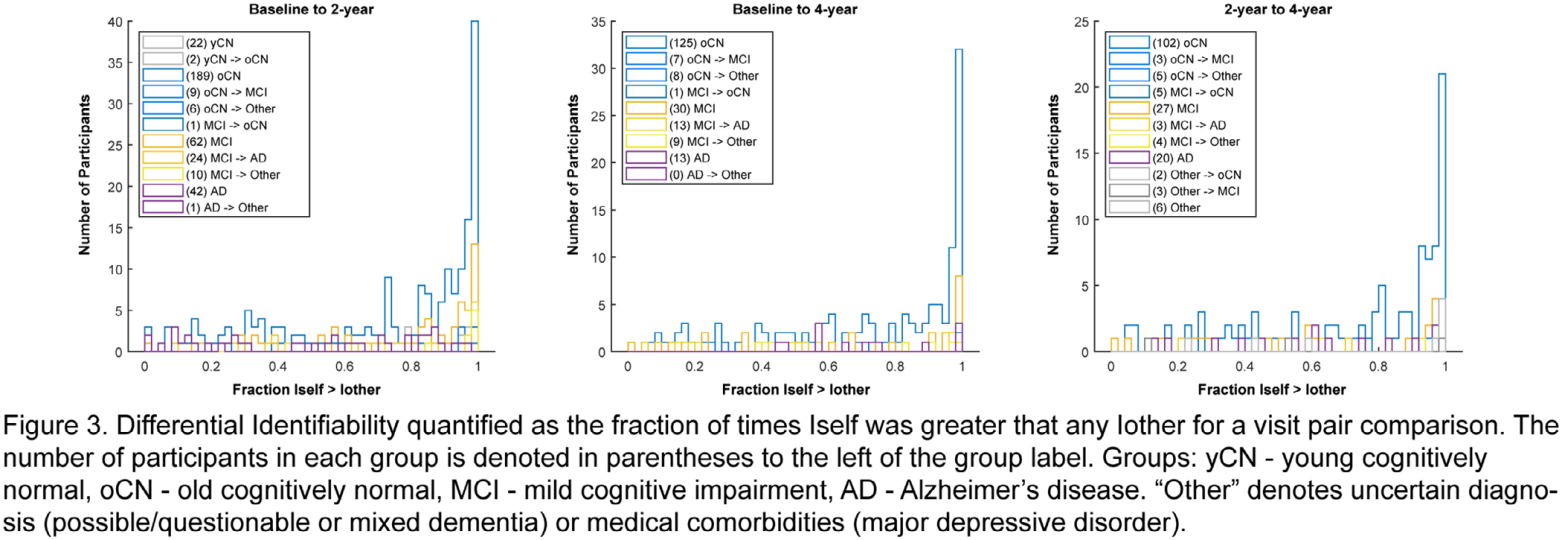# Functional connectivity network identifiability across a multi‐year follow‐up in the Korean Brain Aging Study for the Early Diagnosis and Prediction of AD

**DOI:** 10.1002/alz70862_110033

**Published:** 2025-12-23

**Authors:** Evgeny J. Chumin, Enrico Amico, Sahith Peddireddy, Dahyun Yi, Min Soo Byun, Jun‐Young Lee, Yu Kyeong Kim, Koung Mi Kang, Chul‐Ho Sohn, Shannon L Risacher, Yu‐Chien Wu, Olaf Sporns, Kwangsik Nho, Andrew J. Saykin, Dong Young Lee

**Affiliations:** ^1^ Indiana Alzheimer’s Disease Research Center, Indiana University School of Medicine, Indianapolis, IN USA; ^2^ Center for Neuroimaging, Department of Radiology and Imaging Sciences, Indiana University School of Medicine, Indianapolis, IN USA; ^3^ University of Birmingham, Birmingham UK; ^4^ Indiana Alzheimer's Disease Research Center, Indiana University School of Medicine, Indianapolis, IN USA; ^5^ Seoul National University Medical Research Center, Seoul Korea, Republic of (South); ^6^ Department of Psychiatry, Seoul National University College of Medicine, Seoul Korea, Republic of (South); ^7^ Department of Neuropsychiatry, SMG‐SNU Boramae Medical Center, Seoul Korea, Republic of (South); ^8^ SMG‐SNU Boramae Medical Center, Seoul Korea, Republic of (South); ^9^ Seoul National University College of Medicine, Seoul Korea, Republic of (South); ^10^ Department of Radiology, Seoul National University Hospital, Seoul Korea, Republic of (South); ^11^ Department of Radiology and Imaging Sciences, Indiana University School of Medicine, Indianapolis, IN USA; ^12^ Department of Psychological and Brain Sciences, Indiana University, Bloomington, IN USA; ^13^ Department of Radiology and Imaging Sciences, Indiana Alzheimer’s Disease Research Center, Center for Neuroimaging, Indiana University School of Medicine, Indianapolis, IN USA; ^14^ Center for Neuroimaging, Department of Radiology and Imaging Sciences, Indiana University School of Medicine, Indianapolis, IN USA; ^15^ Department of Medical and Molecular Genetics, Indiana University School of Medicine, Indianapolis, IN USA; ^16^ Center for Neuroimaging, Indiana University School of Medicine, Indianapolis, IN USA

## Abstract

**Background:**

Resting state functional magnetic resonance imaging (rsfMRI) is a promising potential biomarker for diagnostic and prognostic assessment in Alzheimer’s disease (AD) as it provides spatial and temporal information on brain functional connectivity (FC). The protracted course of AD necessitates a better understanding of the longitudinal utility of FC. Therefore, we utilized FC identifiability (ability to match scans at different visits from the same patient) to investigate whether participants with varying diagnostic AD severity displayed differential identifiability over two‐ and four‐year follow‐ups.

**Method:**

KBASE rsfMRI data from 70 younger and 284 older cognitively normal (yCN, mean age: 38±9.8yo and oCN, 69±8yo), 147 mild cognitive impairment (MCI, 73.5±6.9yo), and 87 AD dementia (72.5±7.8yo) participants at baseline, underwent standard preprocessing and nuisance regression to generate Pearson correlation FC networks with the Schaefer 200 cortical region functional parcellation. Identifiability (the correlation between FC connections (edges) for any scan pair) was computed within visit (baseline: *n* = 594, 2‐year: *n* = 371, 4‐year: *n* = 207) and across visit pairs (baseline‐2year: *n* = 369, baseline‐4year: *n* = 207, 2year‐4year: *n* = 181), where participants were further stratified based on visit‐to‐visit change in diagnosis. Measures of interest were self‐identifiability (Iself), mean identifiability to others (Iothers), and differential identifiability defined as the proportion of Iself>Iothers.

**Result:**

Cross‐sectional identifiability at diagnostic group average showed a reduction in AD versus the oCN and MCI groups, white participant level matrices showed variability both within and between diagnostic groups (Figure 1). Longitudinal identifiability recapitulated this variability, while showing that a large portion of participants can be matched based on FC at two and four years apart (Figure 2). Success rate of identifiability was >80% for vast majority oCN participants and was reduced and more variable in diagnostic MCI/AD groups (Figure 3).

**Conclusion:**

The observed variability in identifiability recapitulates prior knowledge in the field at a 2‐year gap, extending for the first time to a 4‐year gap. The variable differential identifiability success rates in diagnostic groups necessitates further investigations into contributing patient specific factors, as explaining this variance is key in furthering the clinical utility of FC in AD.